# Evaluation of menstrual irregularities following COVID-19 infection or vaccination: The impact of COVID anxiety and associated risk factors

**DOI:** 10.1097/MD.0000000000038771

**Published:** 2024-06-28

**Authors:** Halime Seda Küçükerdem, Tuğçe Doğa Özdemir

**Affiliations:** a Department of Family Medicine, Health Science University, Izmir Bozyaka Education and Research Hospital, Izmir, Turkey; b Department of Radiology, Health Science University, Izmir Bozyaka Education and Research Hospital, Izmir, Turkey.

**Keywords:** anxiety, coronavirus disease 2019 infection, menstrual irregularities

## Abstract

There has been significant interest in the changes in menstrual cycles following coronavirus disease 2019 (COVID-19) infection or vaccination. This study aimed to investigate the evidence for such changes and their potential risk factors. We used a descriptive study design and gathered data by sharing an online survey questionnaire on social media platforms. The questionnaire included questions regarding sociodemographic factors, menstrual cycle changes, and COVID-19 anxiety. The study population comprised women aged 18 to 49 years from Izmir. All data analyses were performed using Statistical Package for the Social Sciences 21.0. The risk factors influencing menstrual irregularities were determined after the COVID binary logistic regression analysis, including univariate and multivariate models. Among the 465 participants, those with an associate’s degree had a significantly higher risk of menstrual irregularities than those with a high school diploma (*P* = .012). Anxiety scores emerged as a significant risk factor for menstrual cycle irregularities (*P* = .026). However, neither COVID-19 infection nor vaccination resulted in significant changes in the menstrual cycle characteristics (*P* > .05). Other sociodemographic variables, such as age, body mass index, and smoking, were not significantly associated with menstrual cycle changes(*P* > .05). The study findings suggest that educational level and anxiety may play a role in menstrual irregularities, whereas COVID-19 infection or vaccination itself may not directly affect menstrual cycle.

## 1. Introduction

The coronavirus disease 2019 (COVID-19) outbreak, which originated in Wuhan, China, in December 2019, quickly became a pandemic affecting the lives of billions of people.^[1–4]^ In addition to being a physical health problem, it has psychological, economic, and political effects worldwide.^[1,2]^ As of August 2022, >598 million confirmed cases and over 6.4 million deaths had been reported globally.^[5]^

Owing to the rapid spread of the virus and the lack of proven treatment, the United States Food and Drug Administration granted emergency authorization for Pfizer/BioNTech and Moderna vaccines on December 11 and 18, 2020, respectively.^[6]^ The Pfizer/BioNTech vaccine has been approved for emergency use within the shortest time available.^[6,7]^ Many individuals have tried to protect themselves and others using these rapidly approved vaccines. However, as with many new vaccines or medications, concerns and myths have also emerged. Attitudes towards vaccination vary across communities and are influenced by numerous external factors. For example, studies have shown significant relationships between sex, educational level, history of influenza vaccination, and vaccination rate.^[8,9]^ Another study indicated that attitudes towards vaccination are affected by individuals’ lifestyles, health perceptions, beliefs about childhood diseases, perceptions of disease risks, perceptions of vaccine efficacy and components, and trust in institutions.^[10]^

The ability to encourage vaccination is equally important in vaccine development. This significant public health problem requires each community to implement its own policies.^[11]^ According to previous studies, the most common concerns related to vaccination are “safety issues related to the vaccine development process and “potential side effects.”^[12,13]^ With these newly approved vaccines under emergency use authorization, there have been concerns about the unknown long-term side effect profile and the lack of sufficient scientific studies, particularly regarding the possible impact on reproductive health in adults of reproductive age.^[14]^

In outpatient clinic applications, patients have reported menstrual cycle changes following COVID-19 infection or vaccination, raising the issue of a possible relationship. A literature review revealed various studies conducted in different countries on this issue.^[15,16]^ An increasing number of reports on changes in menstrual cycles following COVID-19 vaccination and infection have led to the speculation that COVID-19 infection may directly or indirectly contribute to menstrual irregularities.^[17,18]^

Anxiety typically arises in response to stressful events and can affect an individual’s quality of life.^[19]^ During the pandemic, an increased prevalence of anxiety disorders was observed in the general population,^[20]^ and the literature has indicated an association between anxiety and menstrual irregularities.^[21]^

Family doctors play a crucial role in offering preventive health care, and immunization programs are a vital part of this. The need to effectively communicate with patients and initiate individualized vaccination efforts highlights the significant role of family physicians in disease prevention. Similar to other infectious diseases, family physicians play a crucial role in vaccination campaigns during the fight against the COVID-19 pandemic.

Considering the diverse sociodemographic and genetic characteristics of our society, it is necessary to conduct a study to investigate whether there is any evidence of changes in menstrual cycles following vaccination or COVID-19. Our study is a pioneering effort to understand the effects of COVID-19-related anxiety on menstrual irregularities. This research utilizes a novel sociodemographic and health anxiety questionnaire to measure the direct impacts of pandemic-induced psychological stress on menstrual health.

## 2. Materials and methods

This is a descriptive study. The study population consisted of women aged 18 to 49 years in Izmir Province. According to the 2018 data from the Izmir Governorate, the total number of women residing in Izmir was 2167,934. The sample size of the study was determined to be 384 individuals using the formula for an unknown population at a 95% confidence level and 5% margin of error. Efforts were made to obtain a minimum of 384 animals per treatment group. The questionnaire used in this study is specially designed to measure participants’ levels of COVID-19 anxiety and its potential impacts on menstrual health, which directly links pandemic-related anxiety measurements with menstrual health parameters. After obtaining ethical approval, the researchers shared the Google Survey questionnaire online through social media accounts (LinkedIn, Facebook, Twitter, and Telegram). Responses to the questionnaire were collected between June 15, 2022, and October 15, 2022.

This study adhered to the principles outlined in the Declaration of Helsinki of the World Medical Association and no personal data were included. Ethical approval was obtained from the Non-Interventional Research Ethics Committee of Izmir Bozyaka Education and Research Hospital (Grant no. 2022/95; date: June 8, 2022).

Data were transferred to a computer using the Statistical Package for the Social Sciences (SPSS) version 21.0 software package (IBM Corporation, Armonk). Descriptive statistics such as numbers and percentages were calculated for sociodemographic characteristics, and parametric or nonparametric analysis methods were used based on tests for normal distribution and equal variances. Frequency and percentage distributions were analyzed using the chi-square test and independent *t* test for group comparisons. Differences were considered statistically significant at *P* < .05. The risk factors influencing menstrual irregularities were determined after the COVID binary logistic regression analysis, including univariate and multivariate models.

## 3. Results

### 3.1. Participant characteristics

**Figure 1.**
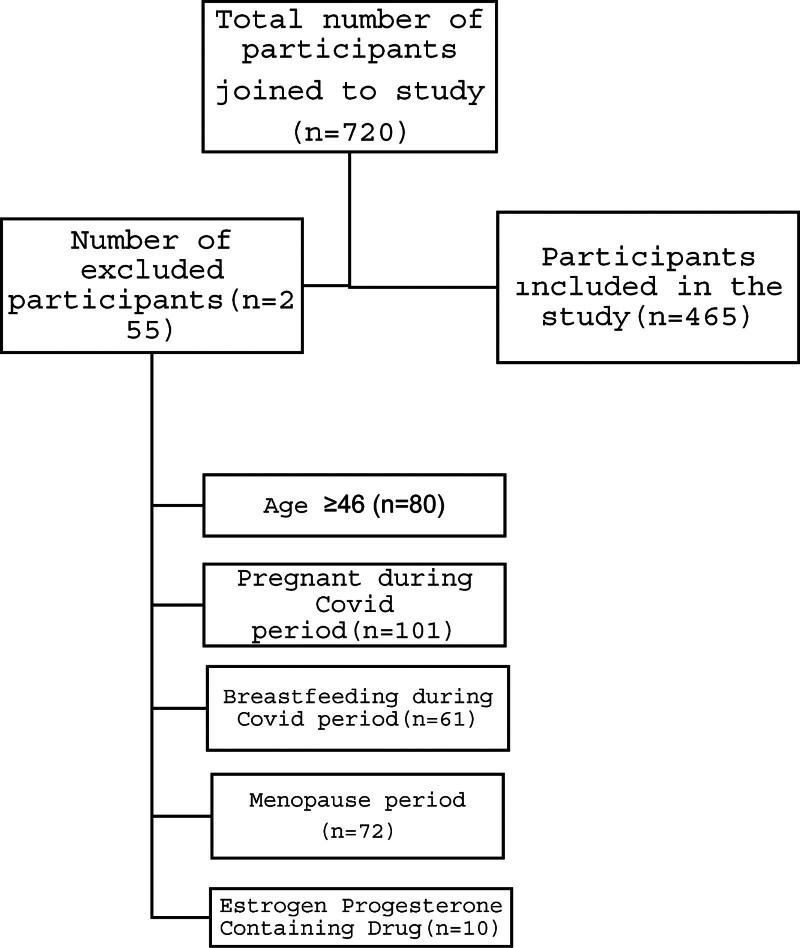
Inclusion and exclusion status of participants in the study. This flowchart illustrates the participant selection process for the study. Initially, 720 participants joined the study. Out of these, 255 were excluded based on specific criteria: 80 were above the age of 46, 101 were pregnant during the COVID period, 61 were breastfeeding during the COVID period, 72 were in the menopause period, and 10 were taking estrogen-progesterone-containing drugs. The remaining 465 participants were included in the study.

Figure 1 presents a diagram illustrating the inclusion and exclusion statuses of the study participants.

The average age of the 465 participants was 34 years (range 18–45 years). Among the participants, 19.4% had completed an educational level of high school or lower and 11.2% had completed an association degree. The majority (69.4%) were university graduates with a master’s or doctoral degree. Additionally, 44.8% of participants had never been affected by COVID-19.

### 3.2. Relationship of independent variables with menstrual irregularities

The general risk factors influencing menstrual irregularities after COVID-19 were examined using binary logistic regression analysis, including univariate and multivariate models. According to the univariate model, individuals with an associate’s degree had a 3.57 times greater risk of menstrual irregularities than those with a high school diploma (*P* = .012). Anxiety score had no statistically significant effect on menstrual irregularities (*P* = .090). Similarly, the other variables included in the univariate model had no significant effect on menstrual cycle irregularities (*P* > .050). When examining the multivariate model results, individuals with an associate’s degree had a 3.435 times greater risk of menstrual irregularities than those with a high school diploma (*P* = .047). The other variables showed no significant effects (*P* > .050) (Table 1).

**Table 1:** Examination of general risk factors influencing menstrual irregularities.

	Regular	Irregular	Total	Univariate		Multivariate	
				OR (95% CI)	*P*	OR (95% CI)	*P*
Age	34.01 ± 7.27	32.88 ± 7.36	33.78 ± 7.3	0.979 (0.945–1.014)	.236		
	35 (15–45)	33 (18–45)	34 (15–45)				
BMI	23.91 ± 4.83	23.47 ± 4.68	23.82 ± 4.8	0.98 (0.924–1.041)	.515		
	23.09 (15.59–43.5)	22.72 (13.71–33.91)	23.03 (13.71–43.5)				
Last school graduated from							
Elementary and middle school	9 (100)	0 (0)	9 (2.6)				
High school	51 (86.4)	8 (13.6)	59 (16.8)	Reference			
Associate degree	25 (64.1)	14 (35.9)	39 (11.2)	3.57 (1.324–9.624)	**.012**	3384 (0.999–11.462)	.050
Bachelor degree	130 (78.8)	35 (21.2)	165 (47.3)	1.716 (0.746–3.95)	.204	2165 (0.762–6151)	.147
Master’s degree/PhD	61 (79.2)	16 (20.8)	77 (22.1)	1.672 (0.662–4.224)	.277	1732 (0.534–5616)	.360
Regularly working							
Yes	206 (78.3)	57 (21.7)	263 (75.4)	1.211 (0.653–2.244)	.544	1094 (0.493–2429)	.825
No	70 (81.4)	16 (18.6)	86 (24.6)	Reference			
Having had COVID-19							
No. I have not had it	133 (83.1)	27 (16.9)	160 (44.8)	Reference			
Yes. I had it	151 (76.6)	46 (23.4)	197 (55.2)	1.501 (0.884–2.548)	.133	1636 (0.893–2998)	.111
Presence of chronic disease							
Yes	50 (79.4)	13 (20.6)	63 (17.6)	1.014 (0.517–1.987)	.968	1583 (0.731–3425)	.244
No	234 (79.6)	60 (20.4)	294 (82.4)	Reference			
Smoking							
Yes	91 (84.3)	17 (15.7)	108 (30.9)	0.617 (0.339–1.122)	.114	0.579 (0.291–1152)	.119
No	185 (76.8)	56 (23.2)	241 (69.1)	Reference			
Use of birth control pills, hormonal IUD, or hormone treatment related to reproductive health during or currently in COVID pandemic							
No	3 (60)	2 (40)	5 (1.4)	Referans			
Yes	281 (79.8)	71 (20.2)	352 (98.6)	0.379 (0.062–2.311)	.293	0.365 (0.046–2873)	.338
Vaccinated against COVID-19							
Yes	272 (79.5)	70 (20.5)	342 (95.8)	1.029 (0.283–3.748)	.965	0.845 (0.199–3577)	.818
No	12 (80)	3 (20)	15 (4.2)	Reference			
Coronavirus anxiety	7.23 ± 3.31	8.04 ± 4.4	7.4 ± 3.58	1.059 (0.991–1.131)	.090	1078 (1003–1159)	**.041**
	6 (5–25)	6 (5–25)	6 (5–25)				
Vaccines applied for immunity against COVID-19							
Only Sinovac	25 (71.4)	10 (28.6)	35 (10.2)	Reference			
Only Biontech	156 (80.4)	38 (19.6)	194 (56.7)	0.609 (0.27–1.375)	.233		
Sinovac and Biontech	91 (80.5)	22 (19.5)	113 (33)	0.604 (0.254–1.441)	.256		
Anxiety							
Low anxiety	209 (80.1)	52 (19.9)	261 (75.9)	Reference			
High anxiety	62 (74.7)	21 (25.3)	83 (24.1)	1.361 (0.762–2.433)	.298		
Age group							
18–35	144 (77)	43 (23)	187 (53.6)	Reference			
36–45	132 (81.5)	30 (18.5)	162 (46.4)	0.761 (0.451–1284)	.306	0.821 (0.446–1509)	.525
							
BMI groups							
Underweight (<18.5)	21 (72.4)	8 (27.6)	29 (9.1)	Reference			
Normal weight (18.5)	153 (80.5)	37 (19.5)	190 (59.9)	0.635 (0.261–1546)	.317	0.603 (0.235–1547)	.292
Overweight	49 (80.3)	12 (19.7)	61 (19.2)	0.643 (0.229–1801)	.401	0.696 (0.229–2113)	.522
Obese	31 (83.8)	6 (16.2)	37 (11.7)	0.508 (0.154–1678)	.267	0.553 (0.158–1.94)	.355

BMI = body mass index, CI = confidence interval, COVID-19 = coronavirus disease 2019, IUD = intrauterine device, OR = odds ratio.

The risk factors influencing menstrual irregularities after COVID-19 were examined using a multivariate model that included age, body mass index, COVID anxiety score, and vaccine administration. Statistical analysis revealed that the COVID anxiety score had a significant effect on menstrual cycle irregularities. An increase of 1 unit in the anxiety score increased the risk of menstrual irregularities by 1.081 times (*P* = .026). However, other variables showed no significant effects (*P* > .050) (Table 2).

**Table 2:** Examination of risk factors influencing menstrual irregularities after COVID-19.

	Regular	Irregular	Total	Univariate		Multivariate	
				OR (95% CI)	*P*	OR (95% CI)	*P*
Age	34.01 ± 7.27	32.88 ± 7.36	33.78 ± 7.3	0.979 (0.945–1.014)	.236		
	35 (15–45)	33 (18–45)	34 (15–45)				
BMI	23.91 ± 4.83	23.47 ± 4.68	23.82 ± 4.8	0.98 (0.924–1.041)	.515		
	23.09 (15.59–43.5)	22.72 (13.71–33.91)	23.03 (13.71–43.5)				
Coronavirus anxiety	7.23 ± 3.31	8.04 ± 4.4	7.4 ± 3.58	1.059 (0.991–1.131)	.090	1.081 (1.009–1.158)	**.026**
	6 (5–25)	6 (5–25)	6 (5–25)				
Vaccines for COVID-19 immunity							
Only Sinovac	25 (71.4)	10 (28.6)	35 (10.2)	Reference			
Only Biontech	156 (80.4)	38 (19.6)	194 (56.7)	0.609 (0.27–1.375)	.233	0.675 (0.257–1777)	.427
Sinovac and Biontech	91 (80.5)	22 (19.5)	113 (33)	0.604 (0.254–1.441)	.256	0.759 (0.274–2102)	.596
Age group							
18–35	144 (77)	43 (23)	187 (53.6)	Reference			
36–45	132 (81.5)	30 (18.5)	162 (46.4)	0.761 (0.451–1284)	.306	0.96 (0.533–1.73)	.892
							
BMI groups							
Underweight	21 (72.4)	8 (27.6)	29 (9.1)	Reference			
Normal weight	153 (80.5)	37 (19.5)	190 (59.9)	0.635 (0.261–1546)	.317	0.525 (0.209–1322)	.171
Overweight	49 (80.3)	12 (19.7)	61 (19.2)	0.643 (0.229–1801)	.401	0.535 (0.184–1553)	.250
Obese	31 (83.8)	6 (16.2)	37 (11.7)	0.508 (0.154–1678)	.267	0.432 (0.12–1556)	.199

BMI = body mass index, CI = confidence interval, COVID-19 = coronavirus disease 2019, OR = odds ratio.

### 3.3. Relationship of menstrual cycle variables with anxiety status

Contracting COVID-19 or receiving COVID-19 vaccine had no impact on the onset of menstrual bleeding (*P* = .473). Similarly, COVID-19 or vaccination did not alter the duration of menstrual bleeding (*P* = .29). There was no significant change in the amount of menstrual bleeding related to COVID-19 status (*P* = .879). Therefore, the return to a normal menstrual cycle after COVID-19 or vaccination was independent of the COVID-19 status (*P* = .429) or employment in a regular job (*P* = .205).

Individuals with chronic illnesses experienced no changes in the timing, duration, or amount of menstrual bleeding after receiving the COVID-19 vaccine or contracting the disease (*P* = .538, *P* = .665, and *P* = .914, respectively) (Table 3).

**Table 3:** Comparison of menstrual cycle variables after COVID-19 infection or vaccination according to anxiety status.

	Anxiety		Total	Test stat.	*P*
	Low anxiety	High anxiety			
How many days apart is the menstrual bleeding after COVID-19 vaccination or disease?					
<21 d	13 (25)	6 (28.6)	19 (26)	1.421	.922
21–25 d	11 (21.2)	3 (14.3)	14 (19.2)		
26–30 d	6 (11.5)	3 (14.3)	9 (12.3)		
31–35 d	8 (15.4)	2 (9.5)	10 (13.7)		
>35 d	13 (25)	6 (28.6)	19 (26)		
Unchanged	1 (1.9)	1 (4.8)	2 (2.7)		
Duration of menstrual bleeding after COVID-19 disease or vaccination					
<2 d	7 (13.5)	1 (4.8)	8 (11)	1.522	.677
2–8 d	27 (51.9)	12 (57.1)	39 (53.4)		
>8 d	9 (17.3)	3 (14.3)	12 (16.4)		
Unchanged	9 (17.3)	5 (23.8)	14 (19.2)		
Change in amount of menstrual bleeding after COVID-19 disease or vaccination					
Less than normal	20 (38.5)	7 (33.3)	27 (37)	0.172	.918
No change	18 (34.6)	8 (38.1)	26 (35.6)		
More than normal	14 (26.9)	6 (28.6)	20 (27.4)		
Regularity of menstrual cycles after COVID-19 vaccine or disease					
Yes, it returned to normal after 1 cycle	4 (7.7)	3 (14.3)	7 (9.6)	9.721	.084
Yes, it returned to normal after 1–3 cycles	12 (23.1)	3 (14.3)	15 (20.5)		
Yes, it returned to normal after 3–6 cycles	1 (1.9)	3 (14.3)	4 (5.5)		
Yes, it returned to normal after 6–12 cycles	1 (1.9)	2 (9.5)	3 (4.1)		
It hasn’t returned to normal yet	29 (55.8)	10 (47.6)	39 (53.4)		
My menstrual cycle did not change	5 (9.6)	0 (0)	5 (6.8)		

COVID-19 = coronavirus disease 2019.

“Chi-square test, frequency (percentage)”.

Similarly, no differences were found in the timing, duration, amount, or return to normal menstrual cycles of menstrual bleeding after COVID-19 vaccination or at the disease onset. No significant association was found between smoking and the timing (*P* = .391), duration (*P* = .829), amount (*P* = .215), or return to normal cycles (*P* = .383) of menstrual bleeding. Details are presented in Table 3.

## 4. Discussion

Individuals with college degrees had a significantly higher risk of menstrual irregularities than those with high school degrees. This finding suggests that education level may play a determining role in menstrual cycle irregularities. Better-educated women may be more aware of their daily routines. According to Kwak et al, “The majority of participants had a high school degree or better, and most households had an income above medium-high, and also they reported that women with irregular menstruation had a lower educational level than those with regular menstruation.,” while the less, taking educational level into account, found that women in the top-income quartile had a higher likelihood of experiencing irregular menstruation.^[22]^

Most studies have reported that in developed countries, obesity and socioeconomic status typically have an inversely proportional relationship.^[23,24]^ According to Egemen, the logistic regression results indicate that women are twice as likely to be obese as men, whereas married people are twice as likely to be obese as single people. A strong negative relationship was found between the likelihood of being overweight or obese and educational level and physical activity. Specifically, as the number of days per week with at least 10 minutes of walking increased, the likelihood of individuals developing class III obesity was reduced by up to half. As for economic status variables, greater household income increases the likelihood of being overweight or obese. In line with studies conducted in developing countries, specifically Turkey, rising income levels are predicted to increase total energy and fat intake, leading to a greater risk of overweight or obesity.^[25]^ Therefore, menstrual cycle irregularities are becoming increasingly problematic.

Anxiety scores were found to increase the risk of menstrual irregularities, with each additional unit increasing the risk by 1.081 times (*P* = .026). This result suggests that anxiety may have a potential impact on hormonal balance and, thus, on the menstrual cycle. Anxiety can trigger the release of stress hormones, leading to hormonal imbalances.^[26–28]^ This can affect the menstrual cycle and result in irregularities.

COVID-19 and vaccination status did not alter the duration of menstrual bleeding (*P* = .29). Previous studies on HPV and flu vaccinations have demonstrated that these can be related to modifications in the menstrual cycle characteristics. However, evidence-based research on COVID-19 vaccination and modifications in menstrual cycle characteristics is not clear.^[29]^

Returning to a normal menstrual cycle after COVID-19 or vaccination was not associated with COVID-19 status (*P* = .429) or regular employment status (*P* = .205). Alvergne et al reported that “18% of the premenopausal participants who received the COVID-19 vaccine (n = 4989) reported alterations in their menstrual cycle after receiving the first dose.” In the same study, it was revealed that “In a second sample of participants (n = 12, 579), both vaccinated and unvaccinated, abnormal menstrual cycle parameters were not linked to COVID-19 vaccination alone, but a history of COVID-19 disease was linked to a higher risk of reporting heavier bleeding, ‘missed’ periods, and intermenstrual bleeding.”^[30]^ In our study, individuals with chronic illnesses did not experience any changes in the onset, duration, or amount of menstrual bleeding after receiving the COVID-19 vaccine or after recovery from the disease. This is similar to the findings of Darney et al and Bouchard et al.^[31,32]^

No significant differences were found in terms of the onset, duration, amount, or return to normal menstrual cycles of menstrual bleeding after COVID-19 vaccination or disease, in relation to smoking. This was not the same finding with Alvergne et al “According to the univariable analysis, the use of contraceptives, cigarette smoking, COVID-19 disease history, and menstrual cycle changes are all related to reporting any changes to menstrual cycles following COVID-19 immunization.”^[30]^

Our findings demonstrate significant effects of pandemic-induced anxiety on menstrual health, underscoring the importance of anxiety management in the design of health policies and interventions.

### 4.1. Limitations of the study

This study has limitations, including the sample’s size and geographic scope. The sample size was limited to 465 individuals, which might restrict the generalizability of our findings to the entire population. Since the research was conducted with participants from only the Izmir region, caution should be exercised regarding the applicability of the findings to the general population of Turkey or other geographic areas. Our study’s sample group was not homogeneously distributed across categories such as education and working life.

The data collection questionnaire required participants to provide responses based solely on their perceptions and memories, possibly leading to recall bias and subjective issues.

Participants were contacted through social media platforms. This method excluded those without internet access and those who did not use social media platforms, which could have affected the results.

### 4.2. Recommendations for future research

Future studies should include participants from various geographic regions to obtain a broader and more diversified sample. Diversifying data collection methods could enhance the reliability and generalizability of the research findings. Specifically, adopting non-survey data collection techniques (e.g., in-depth interviews) and a multi-center approach can expand the scope of the study and yield more comprehensive results.

## 5. Conclusions

These results indicate that COVID-19 infection and vaccination did not directly affect the menstrual cycle. However, it has been determined that indirect factors such as stress and anxiety may have influenced individuals’ menstrual cycles during the pandemic. Therefore, psychological support may have been crucial in managing menstrual cycles in women during the pandemic.

This study has helped us better understand the effects of the COVID-19 pandemic on menstrual cycle irregularities. However, further studies conducted on a larger scale and in different populations are needed to generalize these findings and develop a more comprehensive understanding.

## Author contributions

**Conceptualization:** Halime Seda Kucukerdem, Tuğçe Doğa Özdemir.

**Data curation:** Halime Seda Kucukerdem, Tuğçe Doğa Özdemir.

**Formal analysis:** Halime Seda Kucukerdem.

**Funding acquisition:** Halime Seda Kucukerdem.

**Investigation:** Halime Seda Kucukerdem.

**Methodology:** Halime Seda Kucukerdem, Tuğçe Doğa Özdemir.

**Resources:** Halime Seda Kucukerdem, Tuğçe Doğa Özdemir.

**Software:** Halime Seda Kucukerdem, Tuğçe Doğa Özdemir.

**Supervision:** Halime Seda Kucukerdem.

**Validation:** Halime Seda Kucukerdem.

**Writing – original draft:** Halime Seda Kucukerdem.

**Writing – review & editing:** Halime Seda Kucukerdem.
